# Association between Proximity to a Health Center and Early Childhood Mortality in Madagascar

**DOI:** 10.1371/journal.pone.0038370

**Published:** 2012-06-04

**Authors:** Saori Kashima, Etsuji Suzuki, Toshiharu Okayasu, Razafimahatratra Jean Louis, Akira Eboshida, S. V. Subramanian

**Affiliations:** 1 Department of Public Health and Health Policy, Hiroshima University Graduate School of Biomedical Sciences, Hiroshima, Japan; 2 Department of Epidemiology, Okayama University Graduate School of Medicine, Dentistry and Pharmaceutical Sciences, Okayama, Japan; 3 HIV Prevention Strengthening Project / Japan International Cooperation Agency Madagascar, Antananarivo, Madagascar; 4 Ministry of Health in Service of Statistic Health, Antananarivo, Madagascar; 5 Department of Society, Human Development, and Health, Harvard School of Public Health, Boston, Massachusetts, United States of America; Tulane University School of Public Health and Tropical Medicine, United States of America

## Abstract

**Objective:**

To evaluate the association between proximity to a health center and early childhood mortality in Madagascar, and to assess the influence of household wealth, maternal educational attainment, and maternal health on the effects of distance.

**Methods:**

From birth records of subjects in the Demographic and Health Survey, we identified 12565 singleton births from January 2004 to August 2009. After excluding 220 births that lacked global positioning system information for exposure assessment, odds ratios (ORs) and their 95% confidence intervals (CIs) for neonatal mortality and infant mortality were estimated using multilevel logistic regression models, with 12345 subjects (level 1), nested within 584 village locations (level 2), and in turn nested within 22 regions (level 3). We additionally stratified the subjects by the birth order. We estimated predicted probabilities of each outcome by a three-level model including cross-level interactions between proximity to a health center and household wealth, maternal educational attainment, and maternal anemia.

**Results:**

Compared with those who lived >1.5–3.0 km from a health center, the risks for neonatal mortality and infant mortality tended to increase among those who lived further than 5.0 km from a health center; the adjusted ORs for neonatal mortality and infant mortality for those who lived >5.0–10.0 km away from a health center were 1.36 (95% CI: 0.92–2.01) and 1.42 (95% CI: 1.06–1.90), respectively. The positive associations were more pronounced among the second or later child. The distance effects were not modified by household wealth status, maternal educational attainment, or maternal health status.

**Conclusions:**

Our study suggests that distance from a health center is a risk factor for early childhood mortality (primarily, infant mortality) in Madagascar by using a large-scale nationally representative dataset. The accessibility to health care in remote areas would be a key factor to achieve better infant health.

## Introduction

A decrease in infant mortality by two-thirds between 1990 and 2015 is one of the 4th Millennium Development Goals [1]. However, many developing countries (primarily in sub-Saharan Africa) have made little or no progress in recent years, and this has been associated with poor universal access to health-care services [1]. Accessibility to health-care facilities is conceptualized as being influenced by several basic elements such as environmental factors, population determinants, or health behavior [2]. Although previous studies have shown that distance to a health center is a primary factor in measuring the accessibility to health services [3]–[6], a recent review concluded that there is no robust evidence of an association between distance to a health-care facility and early childhood mortality [7]. This may be due to the fact that previous studies failed to consider other primary determinants of child health, e.g., financial barriers to health care [7]. To our knowledge, two recent studies from Ethiopia and Kenya focused on the social factors (i.e., maternal educational attainment or household wealth) in their evaluations of the association between access to a health center and childhood health [3], [8].

We therefore sought to evaluate whether the proximity to a health center is associated with neonatal mortality and infant mortality by using a nationally representative sample in Madagascar. Further, we assessed whether these associations are modified by household wealth status, maternal education, or maternal health status.

## Methods

### Subjects

**Figure 1:**
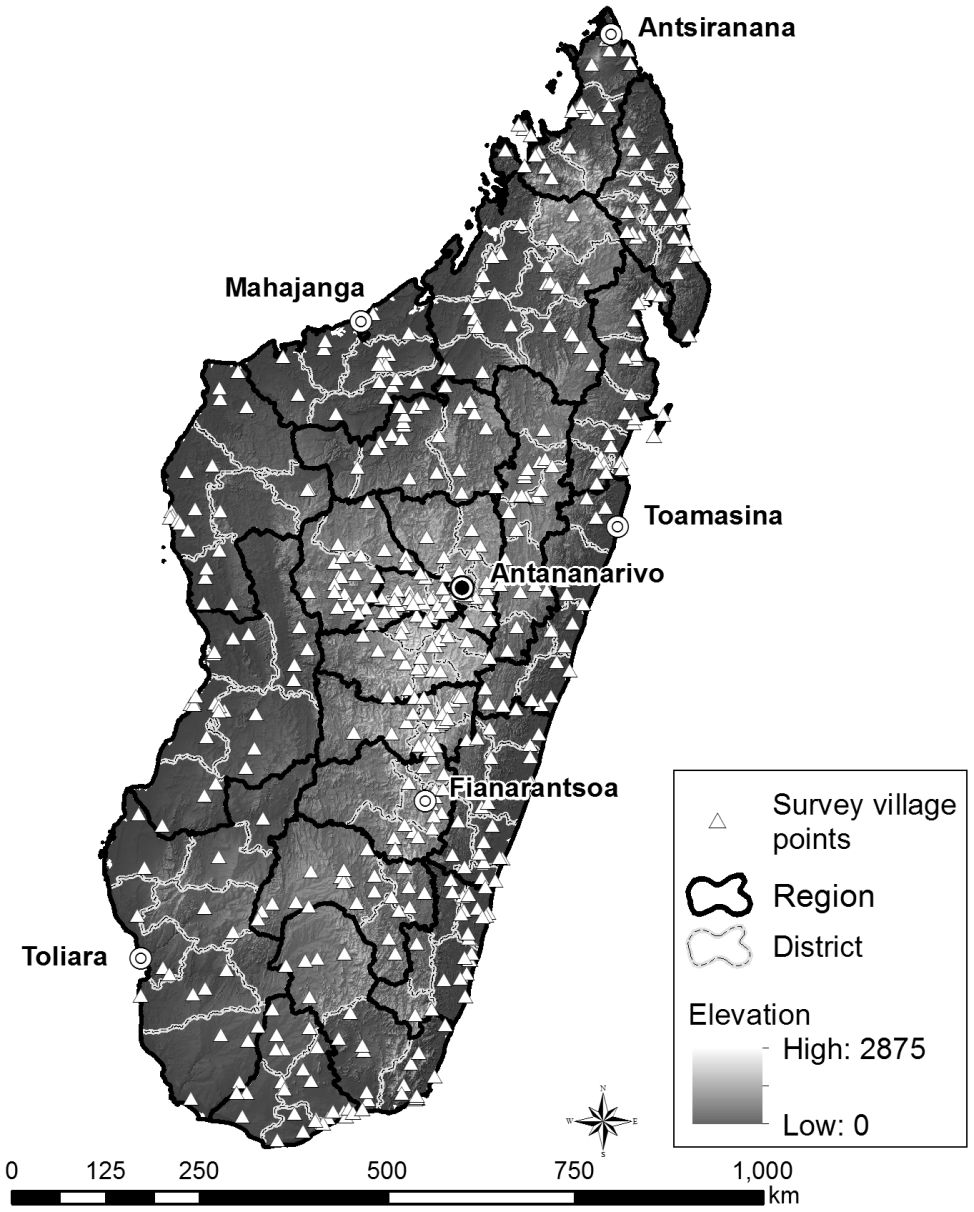
A map of Madagascar indicating the survey points for the 2008–2009 Demographic and Health Survey (EDSMD-IV). DHS, Demographic and Health Survey; EDSMD, quatrième Enquête Démographique et de Santé réalisée à Madagascar.

The study was based on 48464 birth records from the 2008–2009 Demographic and Health Survey (quatrième Enquête Démographique et de Santé réalisée à Madagascar: EDSMD-IV), which is a nationally representative sample survey. The 2008–2009 EDSMD-IV fieldwork was carried out from November 2008 to mid-August 2009 in selected areas in 22 regions in Madagascar (Figure 1). The EDSMD-IV samples were selected using a stratified two-stage design. First, each of the 22 regions was divided into 45 strata designated as urban, rural, and the city of Antananarivo. Then, 600 clusters were identified with a probability proportional to the size of the 22 regions. In the second stage, 32 households were randomly selected from each of the 600 clusters [9]. Each household was then surveyed.

The EDSMD-IV collected demographic, socioeconomic, and health information from each household using three questionnaires: the Household Questionnaire, the Women’s Questionnaire for ever-married women aged 15–49 years, and the Men’s Questionnaire for currently married men aged 15–54 years. In total, 18177 ever-married women were identified in the target area, and complete interviews were obtained with 17375 (96%). On the basis of the Women’s Questionnaire, 48464 birth records were created in the EDSMD-IV.

**Figure 2:**
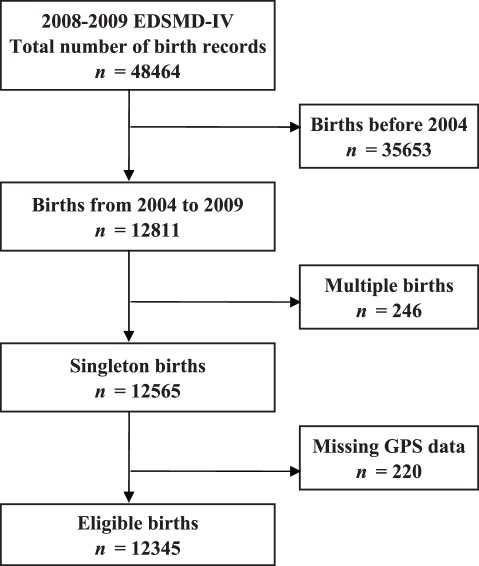
A selection of eligible births from the 2008–2009 EDSMD-IV birth records. GPS, global positioning system; EDSMD, quatrième Enquête Démographique et de Santé réalisée à Madagascar.

In the present study, we extracted details of 12565 singleton births between January 2004 and August 2009 from the birth records, and excluded 220 births that lacked global positioning system (GPS) information for exposure assessment (Figure 2). We thus included 12345 births in the study.

### Health Delivery System in Madagascar

**Figure 3:**
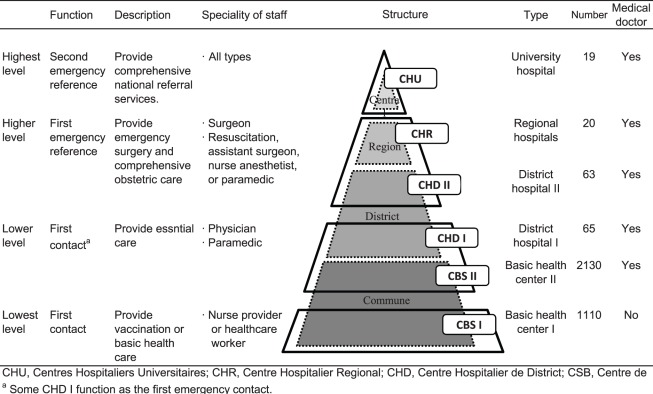
Pyramidal four-stage referral system of health-care delivery in Madagascar.

According to the Plan de Développement Secteur Santé 2007–2011, the health delivery system in Madagascar is composed of four types of health centers: basic health centers (Centre de Santé de Base: CSB) I and II; district hospitals (Centre Hospitalier de District: CHD) I and II; regional hospitals (Centre Hospitalier Regional: CHR); and university hospitals (Centres Hospitaliers Universitaires: CHU). Among these, only CSB I is not assigned a full-time medical doctor. These health facilities are composed of a four-step pyramidal referral system (Figure 3). The majority of these facilities is in imbalances in the distribution of medical staff [10] and concentrated in Antananarivo and other major cities. Indeed, around 28% of doctors serve 75% of the population living in the rural areas [10]. In these public health centers, the basic physical examination was provided without any charge. Also the Madagascar government introduced an equity funds system for achieving universal access to health care. The system funds the basic care or essential drugs to residents if they are certified as being poor by communities (fokontany and commune) [11]. Although these programs were provided by the government, Madagascar ranks the third lowest among the 44 African countries in term of hospital care availability [10].

### Measures

We measured the distance from each household to the nearest health center to assess the subjects’ accessibility to health-care facilities. We included 3309 public health centers from all levels of the pyramidal system (Figure 3), excluding 98 centers because of a lack of location information (latitude and longitude), or absence of type of the health center. A location of these health centers was obtained based on the health care mapping software (Cart Sanitaire Madagascar) from the Service of Statistic Health of the Ministry of Health, which was updated in February 2011 and was supported by the Japan International Cooperation Agency. In the survey, location information of the village or settlement of the household was gathered by a trained interviewer using a GPS. Among the 594 GPS points available in the EDSMD-IV (version 2, updated in April 2011), 585 points could be assigned to the households in the present study. One GPS point represented 21 households on average (maximum, 50; minimum, 5; standard deviation, 8.7). To protect the privacy of respondents, offsets were employed by the EDSMD-IV. The offsets ranged from 0–2 km in urban areas and 0–5 km in rural areas. Furthermore, in rural areas, a 10 km offset was applied to every 100th village. All geographic variables were analyzed using the geographic information systems software ArcGIS (ESRI Japan Inc., version 9.3).

As health outcomes of interest, we used neonatal mortality (<28 days) and infant mortality (<1 year). These were ascertained from the Woman’s Questionnaire in the EDSMD-IV.

In line with previous studies [7], [12]–[14], our covariate sets included child, maternal as well as household characteristics. As the child characteristics, we adjusted for the birth order. The maternal characteristics included maternal age at birth, current maternal smoking status (smoker or not), and birth spacing (<2 years or ≥2 years). Further, to assess current maternal health status, we used maternal anemia (hemoglobin <11.0 g/dL), maternal height, and maternal body mass index. Note that of the 12345 eligible births, the information on maternal health status was available for only 6041 births. The household characteristics included household wealth, maternal education level (no education, primary school, secondary school or higher), religion, type of the nearest health center (CSB I vs. all other), and existence of referral hospital within 30 km, which is assumed to be 1 hour’s travel by car. Household wealth was defined in terms of characteristics related to wealth status, including household ownership of a number of consumer items such as a television and a car, dwelling characteristics such as flooring materials, drinking water source, and toilet facilities [15]. Then, the index was divided into tertiles. As referral hospitals, 102 centers including CHD II, CHR, and CHU in the higher or highest levels of the pyramidal system were adopted (Figure 3).

### Statistical Analysis

We categorized the distance into five groups: first, we adopted two cut-off points (≤5 km, >5–10 km, and >10 km), as recommended by the World Health Organization (WHO) to monitor the health status in developing countries; then, we additionally divided the distance ≤5 km into three categories, approximately by tertile (≤1.5 km, >1.5–3.0 km, and >3.0–5.0 km). We also evaluated linearity of the crude relationship between a distance to a health center and the risk for each outcome in a graphic examination by using linear models (LM). By using the lm function in R version 2.14.1 (R development Core Team 2011), we utilized natural splines with five degrees of freedom for the distance.

The data had a three-level structure of 12345 births at level 1, nested within 584 village locations at level 2, in turn nested within 22 regions at level 3. We thus used three-level logistic regression models with a random intercept. In other words, we allowed the intercept to vary across geographic localities since our data covers the whole nation with wide variations in terms of regional characteristics, e.g., malaria endemicity and different intervention programs for vaccinations. Note that a distance to a health center, type of the nearest health center, and existence of referral hospitals within 30 km were treated as level 2 variables in the models. After examining the crude association between the proximity to a health center and each health outcome, we adjusted for child characteristics, maternal characteristics, and household characteristics (model 1). Then, we additionally adjusted for maternal health status (model 2). The fixed and random parameter estimates (along with their standard errors) for the multilevel binomial logit link model were calibrated using a marginal quasi-likelihood procedure with first order Taylor series expansion, as implemented within the MLwiN 2.24 [16]. We used the second nearest group (>1.5–3 km) as the reference category, because the nearest group (≤1.5 km) was likely to be comprised of an unrepresentative subjects with high wealth status and maternal education attainment who lived in urban areas. We calculated the odds ratios (ORs) and 95% confidence intervals (CIs) for each health outcome. A *P*-value <0.05 (two-sided test) was considered statistically significant.

Although we adjusted for the birth order in the main analysis, a previous study has implied that the association between birth order and childhood mortality might be J- or U-shaped [10]. Thus, we additionally stratified the subjects according to the birth order, and examined the associations between proximity to a health center and each health outcome. In the stratified analyses, the birth order was divided into three categories (first order, second or third order, and fourth or more). As a sensitivity analysis, we also conducted the analyses by using the nearest group (≤1.5 km) as a reference category.

Subsequently, we estimated mean predicted probabilities by a three-level model, including cross-level interaction, between proximity to a health center and household wealth, maternal educational attainment, and maternal health. In this analysis, we modeled the distance as a continuous variable (per increase of 1 km), and we calculated mean predicted probabilities for each health outcome, adjusting for maternal characteristics, maternal health status, and household characteristics.

As a supplementary analysis, we described behavioral characteristics of the subjects and their mothers by the distance to a health center.

## Results

Table 1 shows the demographic characteristics of the subjects, their households, and their mothers by proximity to a health center. We observed a substantial difference in the wealth status of households and maternal educational attainment; in particular, approximately 70% of the nearest group was classified as being the richest. In addition, maternal anemia was more frequent in the most distant area. The results of graphical examinations are shown in Figure S1. In the areas with less than 10 km from a health center, linear relations were observed, but the relations changed at further area than 10 km.

**Table 1:** Demographic characteristics of the subjects, their mothers, and their household by distance from a health center (*n* = 12345).

	*n* for analysis	≤1.5 km		>1.5–3.0 km		>3.0–5.0 km		>5.0–10.0 km		>10 km	
Child characteristics											
singleton birth [no.]	12345	3036		2955		2731		2435		1186	
child sex [no. (%)]	12345										
Male		1565	(51.5)	1508	(51.0)	1417	(51.9)	1206	(49.5)	628	(53.0)
Female		1473	(48.5)	1447	(49.0)	1314	(48.1)	1229	(50.5)	558	(47.0)
birth order [no. (%)]^a^	12345	846	(27.8)	654	(22.1)	595	(21.8)	525	(21.6)	282	(23.8)
First		684	(22.5)	556	(18.8)	482	(17.6)	430	(17.7)	212	(17.9)
Second		508	(16.7)	431	(14.6)	406	(14.9)	353	(14.5)	169	(14.2)
Third		350	(11.5)	396	(13.4)	303	(11.1)	294	(12.1)	146	(12.3)
Fourth		650	(21.4)	918	(31.1)	945	(34.6)	833	(34.2)	377	(31.8)
more than fifth		846	(27.8)	654	(22.1)	595	(21.8)	525	(21.6)	282	(23.8)
Maternal characteristics											
preceding birth interval < 2 years [no. (%)]^a^	12345	400	(13.2)	521	(17.6)	501	(18.3)	494	(20.3)	248	(20.9)
mean age of mothers at birth [year (SD)]	12345	26	(6.9)	26	(7.2)	26	(7.3)	26	(7.4)	25	(6.9)
mother’s smoking [no. (%)]^a^	12340										
Nonsmoker		2737	(90.1)	2565	(86.8)	2389	(87.5)	2116	(86.9)	1037	(87.4)
current smoker		299	(9.8)	390	(13.2)	342	(12.5)	316	(13.0)	149	(12.6)
Maternal health status											
anemia [no. (%)]^a^	6041										
No		955	(31.4)	910	(30.8)	813	(29.8)	730	(30.0)	321	(27.1)
Yes		474	(15.6)	559	(18.9)	547	(20.0)	489	(20.1)	243	(20.5)
not measured		2405	(53.0)	1486	(50.3)	1360	(49.8)	1219	(50.1)	564	(47.6)
height [cm (SD)]	6119	154	(0.6)	153	(0.6)	153	(0.6)	153	(0.6)	154	(5.6)
body mass index (SD)	6111	21	(3.3)	20	(2.8)	20	(2.4)	20	(2.3)	21	(2.4)
Household characteristics											
wealth [no. (%)]a,b	12345										
Highest		2076	(68.3)	1057	(35.8)	583	(21.3)	264	(10.8)	135	(11.4)
Middle		524	(17.2)	1081	(36.6)	1161	(42.5)	939	(38.6)	409	(34.5)
Lowest		438	(14.4)	817	(27.6)	987	(36.1)	1232	(50.6)	642	(54.1)
maternal highest education [no. (%)]^a^	12343										
Higher		132	(4.3)	21	(0.7)	4	(0.1)	3	(0.1)	1	(0.1)
Secondary		1139	(37.5)	581	(19.7)	332	(12.2)	196	(8.0)	79	(6.7)
Primary		1308	(43.1)	1605	(54.3)	1595	(58.4)	1307	(53.7)	560	(47.2)
no education		459	(15.1)	748	(25.3)	800	(29.3)	929	(38.2)	546	(46.0)
religion [no. (%)]^a^	12342										
Catholic		1058	(34.8)	1119	(37.9)	883	(32.3)	581	(23.9)	275	(23.2)
Protestant		1189	(39.1)	1062	(35.9)	742	(27.2)	760	(31.2)	258	(21.8)
other (Muslim, Traditional religion, other)		397	(13.1)	191	(6.5)	211	(7.7)	150	(6.2)	96	(8.1)
no religion		392	(12.9)	582	(19.7)	895	(32.8)	944	(38.8)	557	(47.0)
type of the nearest health center (CSB I) [no. (%)]^a^	12345	340	(11.3)	981	(33.2)	829	(30.4)	577	(23.7)	482	(40.6)
existence of reference hospital within 30 km [no. (%)]^a^	12345	2386	(78.5)	1906	(64.5)	1288	(47.2)	841	(34.5)	107	(9.0)

no., number; SD, standard deviation.

The total number of subjects and percentages may not sum correctly owing to rounding and missing data.

a The percentage is obtained by dividing the number of participants by the total number of births, stratified according to distance to a health center.

b Wealth was calculated based on tertile category of wealth score within country.

Table 2 shows the results of the analysis of the association between distance to a health center and each health outcome. Compared with those who lived >1.5–3.0 km from a health center, the risks for neonatal mortality and infant mortality tended to increase among those who lived further than 5.0 km from a health center; the adjusted ORs for neonatal mortality and infant mortality for those who lived >5.0–10.0 km away from a health center were 1.36 (95% CI: 0.92–2.01) and 1.42 (95% CI: 1.06–1.90), respectively (model 1). Even after additionally adjusting for maternal health status (model 2), the point estimates of these ORs did not substantially change. It is notable that the positive associations were slightly attenuated for those who lived ≥10 km away from a health center. See Table S1 for the results of a sensitivity analysis using ≤1.5 km as a reference.

**Table 2:** Numbers, proportions, and ORs with 95% CIs between proximity to a health center and health outcomes (*n* = 12345).

				Crude model		Adjusted model 1^a^		Adjusted model 2^b^	
	Total *n*	Case *n*	(%)	OR	(95% CI)	OR	(95% CI)	OR	(95% CI)
Neonatal mortality									
≤1.5 km	3038	58	(1.9)	0.94	(0.64–1.38)	0.85	(0.57–1.26)	0.70	(0.40–1.24)
>1.5–3.0 km	2954	61	(2.1)	1	(reference)	1	(reference)	1	(reference)
>3.0–5.0 km	2730	57	(2.1)	1.04	(0.71–1.52)	1.10	(0.75–1.62)	0.98	(0.58–1.66)
>5.0–10.0 km	2435	59	(2.4)	1.21	(0.83–1.76)	1.36	(0.92–2.01)	1.38	(0.82–2.32)
>10 km	1186	24	(2.0)	1.02	(0.62–1.67)	1.21	(0.72–2.05)	1.41	(0.72–2.75)
Infant mortality									
≤1.5 km	3038	107	(3.5)	0.88	(0.66–1.19)	0.92	(0.68–1.24)	0.80	(0.53–1.22)
>1.5–3.0 km	2954	123	(4.2)	1	(reference)	1	(reference)	1	(reference)
>3.0–5.0 km	2730	106	(3.9)	0.98	(0.73–1.31)	0.97	(0.73–1.30)	0.97	(0.66–1.43)
>5.0–10.0 km	2435	133	(5.5)	1.42	(1.07–1.88)	1.42	(1.06–1.90)	1.34	(0.91–1.97)
>10 km	1186	49	(4.1)	1.07	(0.73–1.55)	1.12	(0.75–1.66)	1.18	(0.70–1.97)

OR, odds ratio; CI, confidence interval.

a Adjusted for the birth order, the type of the nearest health center (CSB I vs. all other), existence of reference hospital within 30 km, wealth, maternal education, religion, maternal smoking, maternal age at birth, and birth spacing. (*n* = 12335).

b In addition to model 1, adjusted for maternal health status at time of interview including anemia, height, and maternal body mass index (*n* = 6006).

Table S2 shows the results of the association between distance to a health center and each health outcome stratified by the birth order. Overall, we observed similar tendencies to the results of Table 2, except for a stratum of first birth order. In the strata of second or third birth order and fourth or more birth order, the adjusted ORs for infant mortality were 2.21 (95% CI: 1.30–3.74) and 1.69 (95% CI: 1.11–2.57), respectively, among those who lived >5.0–10.0 km away from a health center (model 1). Note that, in the stratum of second or third birth order, the adjusted OR for neonatal mortality was 2.77 (95% CI: 1.19–6.44) among those who lived >5.0–10.0 km away from a health center (model 1). By contrast, in the stratum of first birth order, no clear association was observed for neonatal mortality and an inverse association was observed for infant mortality. See Table S3 for the results of a sensitivity analysis using ≤1.5 km as a reference.

The mean predicted probabilities for each health outcome associated with distance to a health center, stratified by households’ wealth status or maternal educational attainment, and maternal health status are shown in Figures S2 and S3. Although there were differences in the probabilities according to maternal education, the net effect of all the factors on the outcomes was essentially weak.

As shown in Table S4, the behavioral characteristics of the subjects and their mothers varied by distance to a health center. The rate of vaccination decreased the further the distance to a health center, and mothers living in further areas tended to feel more problems in visiting health centers.

## Discussion

By using a large-scale nationally representative dataset, we evaluated the effects of distance to a health center on child health in Madagascar. We found that risks for early childhood mortality (primarily, infant mortality) tended to increase among those who lived further than 5 km away from a health facility, compared with those who lived >1.5–3.0 km away. The positive associations were more pronounced among the subgroup of the second or third birth order. In further analyses, we evaluated whether the associations were modified by households’ wealth status, maternal educational attainment, or maternal health status. We observed no significant effects of these factors on outcomes.

Our findings suggest 5 km as a possible threshold for the effects of distance from a health center on early childhood mortality in Madagascar. Previous studies on distance and child health in African countries reported that there was a higher risk of adverse health effects among those who lived further than 4 km [17], further than 5 km [18]–[20], further than 6 km [12], or further than 10 km [21] from a health facility. Among these studies, however, only one study categorized distance to a health center into 4 groups [17], and other studies used dichotomized exposure variables, which precluded robust assessment of the threshold of an effect of distance to a health center. To overcome this limitation, we classified the distance into five groups. Generally, in developing countries, women who live closer to health facilities are more likely to be well educated and wealthier, and we observed the consistent findings with regard to households’ wealth status and maternal educational attainment (Table 1). While we entered the wealth index and maternal education attainment as covariates in the multivariate models, a possibility of residual confounding cannot be completely ruled out. We, thus, used the second nearest group as a reference category.

Of note, the present findings are consistent with the WHO guidelines, which recommend monitoring the health status of those who live further than 5 km away from a health center in developing countries. The present findings may also be comparable to another previous study from Burkina Faso [14], which reported that although the risk of childhood mortality was not increased among those who lived closer than a 3-hour walking distance from the closest health facility, the risk was increased among those who lived further than an 8-hour walking distance. Assuming that people can walk 5 km within 1 hour, a 3-hour walking distance corresponds to 15 km, and an 8-hour walking distance to 40 km.

Interestingly, the results varied substantially across the subgroups of the birth order, and we observed a tendency of inverse associations between distance to a health center and early childhood mortality among the first child. Although the explanation for this finding remains unclear, we a posteriori hypothesize that this phenomenon is partly explained by the risk of stillbirths. We note that primiparous women tended to be teenagers; the proportions of teenage pregnancies were 63%, 20%, and 0.7% for the first, second or third, and fourth or more birth orders, respectively. Furthermore, among primiparous women, the proportions of teenage pregnancies were higher in the further areas; they were 53%, 61%, 67%, 74%, and 71% among those who lived ≤1.5 km, >1.5–3.0 km, >3.0–5.0 km, >5.0–10.0 km, and >10.0 km, respectively, away from a health center. It has been reported that teenage pregnancies, particularly primiparous women, are at an increased risk for intrauterine growth restriction, fetal distress, and intrauterine death [22], Also it has been reported that Madagascar has a high intrapartum still birth rate – 46% of stillbirth is estimated to occur in the intrapartum period [23]. Although the information on stillbirths was not available in the present study, this may partly explain the present findings among the first child.

In developing countries, distance-decay effects have been reported [24], [25], which are a phenomenon of decreasing health care utilization with increasing distance from a health center. Although robust information on health center utilization was not available in the present study, maternal behavioral-characteristics such as adherence to risk reduction interventions might be affected by the degree of accessible information about health promotion. In fact, investigation of uptake of vaccination in the subjects found rates of 81%, 72%, 71%, 61%, and 49% among those who lived ≤1.5 km, >1.5–3.0 km, >3.0–5.0 km, >5.0–10.0 km, and >10.0 km, respectively, away from a health center (Table S4). Indeed, in developing countries, it is very likely that those who live far from a health center have poor accessibility to health services, which would lead to an increased risk of early childhood mortality. In line with this, it has been reported that proximity to a health center and financial assistance are the most important two factors of seeking care for the poorest populations [10].

The present study demonstrated that the distance effects were not modified by household wealth status or maternal educational attainment. Although it has been reported that the effect of distance on hospitalization was attenuated by an increase in area-based maternal education [8], the influence of maternal educational attainment on the distance effects was very weak (Figure S2b). Similarly, a recent study from rural Ethiopia reported that household wealth status did not influence childhood mortality or geographic access to health facilities [17]. In Madagascar, although an equity funding system was implemented four years ago for poor people to receive essential drugs, identification rate as indigent remains quite low, varying between 0 and 0.5% of the population [11], [26]. Future studies should examine whether the funding system can reduce modification effects on distance across different wealth status.

There are some limitations in the present study. First, as noted above, offsets of the GPS in the EDSMD-IV may result in exposure misclassification. In addition, GPS data were measured only at a represented place in each cluster, and thus the point does not necessarily reflect the exact location of each household. Furthermore, the distance was calculated according to the Euclidean distance. Thus, the realistic travel time might be longer than the calculated distance. However, such a misclassification would be nondifferential, and the net effect would be biased toward the null [27]. In addition, missing health centers (3% of all health centers) could also introduce misclassification. Since we could not evaluate whether the distribution of missing centers was differential or not, further evaluation is necessary to update the location information of the health centers.

Second, we should note that new health centers were constructed during the study period; the total number of health centers increased from 3106 in 2004 to 3407 in 2009 [28]. We could not obtain the information on the locations of newly-constructed health centers, and it may be likely that more new health centers were constructed in the furthest areas. This might explain the present findings that the positive associations between distance to a health center and early childhood mortality were slightly attenuated in the furthest areas. In addition, functions of health centers could have changed dynamically during the study period, e.g., medical doctors were temporally absent or medical staff cannot access to centers because of heavy rain. Future studies should address potentially varying effects of the functions of health centers.

Finally, residual confounding should be considered. The maternal health status was measured at interview time. Previous studies have shown that anemia during pregnancy was associated with an increased risk of preterm birth or perinatal mortality [29]–[31]. Additional studies are required to evaluate the effect of distance to a health center on early childhood health with consideration of maternal condition in pregnancy.

In conclusion, our study using a nationally representative sample in Madagascar provides evidence that a longer distance to a health center is a risk factor for early childhood mortality (primarily, infant mortality). Although other factors are involved, accessibility to health care among those who live further than 5 km away from a health center may be a key factor to achieve more favorable early childhood health.

## Supporting Information

Table S1 **Numbers, proportions, and ORs with 95% CIs between proximity to a health center and health outcomes for the births which were liveborn singleton births from January 2004 to July 2009 in a sensitivity analysis (** ***n*** **= 12345).**
(PDF)

Table S2 **Numbers, proportions, and ORs with 95% CIs between proximity to a health center and health outcomes for the births which were liveborn singleton births from January 2004 to July 2009 by the birth order (** ***n*** **= 9443).**
(PDF)

Table S3 **Numbers, proportions, and ORs with 95% CIs between proximity to a health center and health outcomes for the births which were liveborn singleton births from January 2004 to July 2009 by the birth order in a sensitivity analysis (** ***n*** **= 9443).**
(PDF)

Table S4 **Behavioral characteristics of the subjects and their mothers varied by distance to a health center for the births which were liveborn singleton births from January 2004 to July 2009 (** ***n*** **= 12345).**
(PDF)

Figure S1 **Distance to a health center and predicted probabilities for neonatal mortality and infant mortality.** Distance to major road was introduced as a natural spline with five degrees of freedom.
(PDF)

Figure S2 **Predicted probabilities for neonatal mortality and infant mortality stratified by households’ wealth status and maternal education.** We modeled the distance to a health center as a continuous variable (per increase of 1 km), and used the logarithmic scale (base 2) for the graphs. We averaged the predicted probability for the group less than 1 km from a health center, and show the probability for distance at 1 km increments.
(PDF)

Figure S3 **Predicted probabilities for neonatal mortality and infant mortality stratified by maternal anemia.** We modeled the distance to a health center as a continuous variable (per increase of 1 km), and used the logarithmic scale (base 2) for the graphs. We averaged the predicted probability for the group less than 1 km from a health center, and showed the probabilities for distance at 1 km increments.
(PDF)
