# Supplementary material for: Association between Proximity to a Health Center and Early Childhood Mortality in Madagascar
Source: PLoS One. 2012 Jun 4;7(6):e38370. doi: 10.1371/journal.pone.0038370 (PMC3366931; doi:10.1371/journal.pone.0038370)
Supplement: Table S1 — Numbers, proportions, and ORs with 95% CIs between proximity to a health center and health outcomes for the births which were liveborn singleton births from January 2004 to July 2009 in a sensitivity analysis ( n = 12345). (PDF) [file pone.0038370.s001.pdf]

Table S1. Numbers, proportions, and ORs with 95% CIs between proximity to a health center and health outcomes for the births which were liveborn singleton births from January 2004 to July 2009 in a sensitivity analysis[a] (n = 12345)

	Total	Case	(%)	Crude		Adjusted model 1[b]		Adjusted model 2[c]	
	n	*n*		OR	(95% CI)	OR	(95% CI)	OR	(95% CI)
Neonatal mortality									
≤1.5 km	3038	58	(1.9)	1	(reference)	1	(reference)	1	(reference)
>1.5–3.0 km	2954	61	(2.1)	1.07	(0.73–1.56)	1.18	(0.79–1.75)	1.43	(0.81–2.53)
>3.0–5.0 km	2730	57	(2.1)	1.11	(0.75–1.62)	1.30	(0.85–1.98)	1.40	(0.76–2.59)
>5.0–10.0 km	2435	59	(2.4)	1.29	(0.88–1.89)	1.60	(1.04–2.47)	1.97	(1.06–3.66)
>10 km	1186	24	(2.0)	1.08	(0.66–1.79)	1.43	(0.81–2.51)	2.01	(0.94–4.27)
Infant mortality									
≤1.5 km	3038	107	(3.5)	1	(reference)	1	(reference)	1	(reference)
>1.5–3.0 km	2954	123	(4.2)	1.13	(0.84–1.52)	1.09	(0.80–1.48)	1.24	(0.82–1.89)
>3.0–5.0 km	2730	106	(3.9)	1.11	(0.82–1.50)	1.06	(0.77–1.46)	1.21	(0.78–1.89)
>5.0–10.0 km	2435	133	(5.5)	1.61	(1.20–2.16)	1.55	(1.12–2.14)	1.66	(1.06–2.61)
>10 km	1186	49	(4.1)	1.21	(0.82–1.77)	1.22	(0.80–1.86)	1.47	(0.83–2.59)

OR, odds ratio; CI, confidence interval.

[a] We conducted a sensitivity analyses by using the nearest group (≤1.5 km) as a reference category.

[b] Adjusted for the birth order, the type of the nearest health center (CSB I vs. all other), existence of reference hospital within 30 km, wealth, maternal education, religion, maternal smoking, maternal age at birth, and and birth spacing. (n = 12335)

[c] In addition to model 1, adjusted for maternal health status at time of interview including anemia, height, and maternal body mass index. (n = 6006)
