# Supplementary material for: Association between Proximity to a Health Center and Early Childhood Mortality in Madagascar
Source: PLoS One. 2012 Jun 4;7(6):e38370. doi: 10.1371/journal.pone.0038370 (PMC3366931; doi:10.1371/journal.pone.0038370)
Supplement: Table S2 — Numbers, proportions, and ORs with 95% CIs between proximity to a health center and health outcomes for the births which were liveborn singleton births from January 2004 to July 2009 by the birth order ( n = 9443). (PDF) [file pone.0038370.s002.pdf]

Table S2. Numbers, proportions, and ORs with 95% CIs between proximity to a health center and health outcomes for the births which were liveborn singleton births from January 2004 to July 2009 by the birth order (*n* = 9443)

	Total	Case	(%)	Crude		Adjusted model 1[a]		Adjusted model 2[b]	
	n	*n*		OR	(95% CI)	OR	(95% CI)	OR	(95% CI)
**First birth order (*n* = 2902)**									
Neonatal mortality									
≤1.5 km	846	27	(3.2)	0.75	(0.43–1.30)	0.80	(0.44–1.44)	0.63	(0.26–1.55)
>1.5–3.0 km	654	28	(4.3)	1	(reference)	1	(reference)	1	(reference)
>3.0–5.0 km	595	21	(3.5)	0.83	(0.46–1.48)	0.76	(0.42–1.37)	0.95	(0.42–2.16)
>5.0–10.0 km	525	16	(3.0)	0.71	(0.38–1.33)	0.66	(0.34–1.25)	0.87	(0.36–2.08)
>10 km	282	6	(2.1)	0.49	(0.20–1.20)	0.48	(0.19–1.23)	0.54	(0.14–2.03)
Infant mortality									
≤1.5 km	846	43	(5.1)	0.81	(0.52–1.26)	0.87	(0.54–1.40)	0.80	(0.41–1.57)
>1.5–3.0 km	654	43	(6.6)	1	(reference)	1	(reference)	1	(reference)
>3.0–5.0 km	595	32	(5.4)	0.83	(0.52–1.34)	0.77	(0.48–1.25)	0.95	(0.49–1.82)
>5.0–10.0 km	525	23	(4.4)	0.66	(0.39–1.12)	0.59	(0.34–1.00)	0.77	(0.38–1.55)
>10 km	282	10	(3.5)	0.53	(0.26–1.09)	0.49	(0.24–1.03)	0.49	(0.18–1.38)
**Second or third birth order (*n* = 4229)**									
Neonatal mortality									
≤1.5 km	1192	11	(0.9)	0.91	(0.38–2.19)	1.12	(0.45–2.83)	0.72	(0.20–2.63)
>1.5–3.0 km	986	10	(1.0)	1	(reference)	1	(reference)	1	(reference)
>3.0–5.0 km	887	14	(1.6)	1.54	(0.67–3.57)	1.69	(0.72–3.95)	0.81	(0.25–2.65)
>5.0–10.0 km	783	18	(2.3)	2.29	(1.03–5.12)	2.77	(1.19–6.44)	3.01	(1.10–8.25)
>10 km	381	6	(1.6)	1.55	(0.54–4.42)	2.04	(0.67–6.24)	3.30	(0.91–12.03)
Infant mortality									
≤1.5 km	1192	21	(1.8)	0.69	(0.38–1.25)	0.93	(0.50–1.73)	1.03	(0.41–2.57)
>1.5–3.0 km	986	25	(2.5)	1	(reference)	1	(reference)	1	(reference)
>3.0–5.0 km	887	29	(3.3)	1.30	(0.75–2.25)	1.24	(0.72–2.16)	1.44	(0.66–3.13)
>5.0–10.0 km	783	44	(5.6)	2.30	(1.39–3.83)	2.21	(1.30–3.74)	2.91	(1.37–6.17)
>10 km	381	14	(3.7)	1.47	(0.75–2.89)	1.37	(0.67–2.77)	2.20	(0.84–5.75)
**Fourth or more birth order (*n* = 5212)**									
Neonatal mortality									
≤1.5 km	1000	20	(2.0)	1.15	(0.62–2.16)	0.92	(0.49–1.74)	0.99	(0.40–2.44)
>1.5–3.0 km	1314	23	(1.8)	1	(reference)	1	(reference)	1	(reference)
>3.0–5.0 km	1248	22	(1.8)	1.03	(0.56–1.88)	1.19	(0.65–2.17)	1.02	(0.44–2.37)
>5.0–10.0 km	1127	25	(2.2)	1.32	(0.73–2.38)	1.73	(0.94–3.19)	1.33	(0.56–3.12)
>10 km	523	12	(2.3)	1.37	(0.66–2.84)	1.98	(0.92–4.26)	2.17	(0.75–6.24)
Infant mortality									
≤1.5 km	1000	43	(4.3)	1.03	(0.66–1.62)	0.98	(0.62–1.55)	0.79	(0.42–1.47)
>1.5–3.0 km	1314	55	(4.2)	1	(reference)	1	(reference)	1	(reference)
>3.0–5.0 km	1248	45	(3.6)	0.89	(0.58–1.38)	0.92	(0.60–1.43)	0.79	(0.45–1.38)
>5.0–10.0 km	1127	66	(5.9)	1.55	(1.03–2.32)	1.69	(1.11–2.57)	1.25	(0.72–2.19)
>10 km	523	25	(4.8)	1.23	(0.72–2.09)	1.54	(0.88–2.72)	1.73	(0.83–3.60)

OR, odds ratio; CI, confidence interval.

[a] Adjusted for the birth order, the type of the nearest health center (CSB I vs. all other), existence of reference hospital within 30 km, wealth, maternal education, religion, maternal smoking, maternal age at birth, and birth spacing. (*n* = 9436)

[b] In addition to model 1, adjusted for maternal health status at time of interview including anemia, height, and maternal body mass index. (*n* = 4569)
