# Supplementary material for: Association between Proximity to a Health Center and Early Childhood Mortality in Madagascar
Source: PLoS One. 2012 Jun 4;7(6):e38370. doi: 10.1371/journal.pone.0038370 (PMC3366931; doi:10.1371/journal.pone.0038370)
Supplement: Table S3 — Numbers, proportions, and ORs with 95% CIs between proximity to a health center and health outcomes for the births which were liveborn singleton births from January 2004 to July 2009 by the birth order in a sensitivity analysis ( n = 9443). (PDF) [file pone.0038370.s003.pdf]

Table S3. Numbers, proportions, and ORs with 95% CIs between proximity to a health center and health outcomes for the births which were liveborn singleton births from January 2004 to July 2009 by the birth order in a sensitivity analysis[a] (*n* = 9443)

	Total	Case	(%)	Crude		Adjusted model 1[b]		Adjusted model 2[c]	
	n	*n*		OR	(95% CI)	OR	(95% CI)	OR	(95% CI)
**First birth order (*n* = 2902)**									
Neonatal mortality									
≤1.5 km	846	27	(3.2)	1	(reference)	1	(reference)	1	(reference)
>1.5–3.0 km	654	28	(4.3)	1.33	(0.77–2.30)	1.25	(0.69–2.26)	1.59	(0.65–3.91)
>3.0–5.0 km	595	21	(3.5)	1.10	(0.61–1.97)	0.96	(0.50–1.84)	1.51	(0.58–3.91)
>5.0–10.0 km	525	16	(3.0)	0.94	(0.50–1.78)	0.82	(0.40–1.70)	1.38	(0.49–3.84)
>10 km	282	6	(2.1)	0.65	(0.26–1.60)	0.61	(0.22–1.64)	0.86	(0.20–3.63)
Infant mortality									
≤1.5 km	846	43	(5.1)	1	(reference)	1	(reference)	1	(reference)
>1.5–3.0 km	654	43	(6.6)	1.24	(0.79–1.94)	1.15	(0.72–1.85)	1.24	(0.64–2.42)
>3.0–5.0 km	595	32	(5.4)	1.03	(0.64–1.65)	0.89	(0.52–1.51)	1.18	(0.57–2.43)
>5.0–10.0 km	525	23	(4.4)	0.82	(0.49–1.39)	0.67	(0.37–1.22)	0.95	(0.43–2.10)
>10 km	282	10	(3.5)	0.66	(0.32–1.34)	0.57	(0.26–1.24)	0.61	(0.20–1.85)
**Second or third birth order (*n* = 4229)**									
Neonatal mortality									
≤1.5 km	1192	11	(0.9)	1	(reference)	1	(reference)	1	(reference)
>1.5–3.0 km	986	10	(1.0)	1.10	(0.46–2.63)	0.89	(0.35–2.24)	1.38	(0.38–5.01)
>3.0–5.0 km	887	14	(1.6)	1.69	(0.75–3.82)	1.50	(0.61–3.68)	1.12	(0.27–4.59)
>5.0–10.0 km	783	18	(2.3)	2.51	(1.16–5.46)	2.47	(1.01–6.05)	4.15	(1.16–14.85)
>10 km	381	6	(1.6)	1.70	(0.61–4.75)	1.82	(0.56–5.86)	4.55	(1.01–20.56)
Infant mortality									
≤1.5 km	1192	21	(1.8)	1	(reference)	1	(reference)	1	(reference)
>1.5–3.0 km	986	25	(2.5)	1.45	(0.80–2.62)	1.07	(0.58–1.99)	0.97	(0.39–2.44)
>3.0–5.0 km	887	29	(3.3)	1.89	(1.06–3.35)	1.33	(0.72–2.48)	1.40	(0.58–3.41)
>5.0–10.0 km	783	44	(5.6)	3.33	(1.95–5.69)	2.37	(1.29–4.34)	2.84	(1.18–6.81)
>10 km	381	14	(3.7)	2.14	(1.07–4.28)	1.47	(0.67–3.19)	2.14	(0.74–6.20)
**Fourth or more birth order (*n* = 5212)**									
Neonatal mortality									
≤1.5 km	1000	20	(2.0)	1	(reference)	1	(reference)	1	(reference)
>1.5–3.0 km	1314	23	(1.8)	0.87	(0.46–1.62)	1.08	(0.58–2.03)	1.01	(0.41–2.50)
>3.0–5.0 km	1248	22	(1.8)	0.89	(0.47–1.67)	1.28	(0.67–2.48)	1.03	(0.39–2.71)
>5.0–10.0 km	1127	25	(2.2)	1.14	(0.62–2.11)	1.88	(0.96–3.65)	1.34	(0.50–3.60)
>10 km	523	12	(2.3)	1.18	(0.56–2.50)	2.14	(0.94–4.88)	2.20	(0.67–7.16)
Infant mortality									
≤1.5 km	1000	43	(4.3)	1	(reference)	1	(reference)	1	(reference)
>1.5–3.0 km	1314	55	(4.2)	0.97	(0.62–1.52)	1.02	(0.65–1.60)	1.27	(0.68–2.37)
>3.0–5.0 km	1248	45	(3.6)	0.87	(0.54–1.37)	0.94	(0.58–1.52)	1.00	(0.51–1.97)
>5.0–10.0 km	1127	66	(5.9)	1.50	(0.97–2.31)	1.72	(1.08–2.75)	1.59	(0.80–3.15)
>10 km	523	25	(4.8)	1.19	(0.68–2.07)	1.57	(0.85–2.90)	2.19	(0.94–5.10)

OR, odds ratio; CI, confidence interval.

[a] We conducted a sensitivity analyses by using the nearest group (≤1.5 km) as a reference category.

[b] Adjusted for the birth order, the type of the nearest health center (CSB I vs. all other), existence of reference hospital within 30 km, wealth, maternal education, religion, maternal smoking, maternal age at birth, and birth spacing. (*n* = 9436)

[c] In addition to model 1, adjusted for maternal health status at time of interview including anemia, height, and maternal body mass index. (*n* = 4569)
