# Supplementary material for: Association between Proximity to a Health Center and Early Childhood Mortality in Madagascar
Source: PLoS One. 2012 Jun 4;7(6):e38370. doi: 10.1371/journal.pone.0038370 (PMC3366931; doi:10.1371/journal.pone.0038370)
Supplement: Table S4 — Behavioral characteristics of the subjects and their mothers varied by distance to a health center for the births which were liveborn singleton births from January 2004 to July 2009 ( n = 12345). (PDF) [file pone.0038370.s004.pdf]

Table S4. Behavioral characteristics of the subjects and their mothers varied by distance to a health center for the births which were liveborn singleton births from January 2004 to July 2009 (*n* = 12345)

	Total	Distance from a health center									
		≤1.5 km		>1.5-3.0 km		>3.0-5.0 km		>5.0-10.0 km		>10 km	
	n	*n*	(%)	*n*	(%)	*n*	(%)	*n*	(%)	*n*	(%)
**Ever had vaccination**	5720										
Yes		1069	(80.8)	950	(72.0)	879	(70.7)	693	(60.8)	342	(49.3)
No		249	(18.8)	359	(27.2)	360	(28.9)	438	(38.4)	352	(50.7)
Don't know		5	(0.4)	10	(0.8)	5	(0.4)	9	(0.8)	0	(0.0)
**Mother visited health facility last 12month**	12331										
Yes		1640	(54.1)	1427	(48.3)	1188	(43.5)	1017	(41.8)	420	(35.5)
No		1394	(45.9)	1526	(51.7)	1540	(56.5)	1415	(58.2)	764	(64.5)
**How mother thought distance to health facility**	6182										
Not a big problem		1053	(70.9)	892	(59.6)	706	(50.5)	467	(37.9)	134	(23.4)
Big problem		432	(29.1)	605	(40.4)	691	(49.5)	764	(62.1)	438	(76.6)
**How mother thought getting permission to go to health facility**	6188										
Not a big problem		1266	(85.0)	1242	(83.0)	1141	(81.7)	1013	(82.3)	445	(77.7)
Big problem		224	(15.0)	255	(17.0)	256	(18.3)	218	(17.7)	128	(22.3)
**Type of bednet(s) child slept under last night**	10968										
Both treated and untreated bednets		0	(0.0)	0	(0.0)	0	(0.0)	1	(0.0)	0	(0.0)
Only treated bednets		1440	(52.9)	1174	(44.5)	1149	(47.3)	1080	(50.8)	531	(50.4)
Only untreated bednets		123	(4.5)	81	(3.1)	87	(3.6)	70	(3.3)	29	(2.8)
No bednet		1161	(42.6)	1381	(52.4)	1191	(49.1)	976	(45.9)	494	(46.9)

no., number

The total number of subjects and percentages may not sum correctly owing to rounding and missing data.
