# Supplementary material for: Association between Proximity to a Health Center and Early Childhood Mortality in Madagascar
Source: PLoS One. 2012 Jun 4;7(6):e38370. doi: 10.1371/journal.pone.0038370 (PMC3366931; doi:10.1371/journal.pone.0038370)
Supplement: Figure S1 — Distance to a health center and predicted probabilities for neonatal mortality and infant mortality. Distance to major road was introduced as a natural spline with five degrees of freedom. (PDF) [file pone.0038370.s005.pdf]

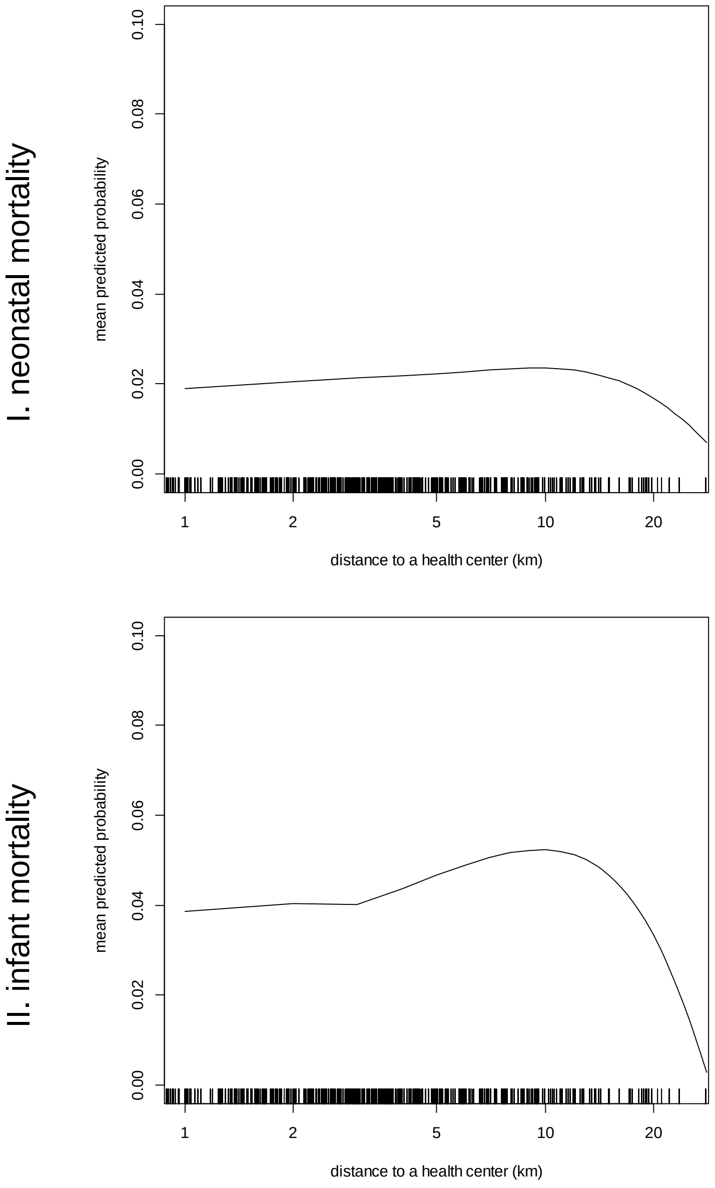

Figure S1. Distance to a health center and predicted probabilities for neonatal mortality and infant mortality.

Distance to major road was introduced as a natural spline with five degrees of freedom. We modeled the distance to a health center as a continuous variable (per increase of 1 km), and used the logarithmic scale (base 2) for the graphs. We averaged the predicted probability for the group less than 1 km from a health center, and showed the probabilities for distance at 1 km increments.
