# Supplementary material for: Association between Proximity to a Health Center and Early Childhood Mortality in Madagascar
Source: PLoS One. 2012 Jun 4;7(6):e38370. doi: 10.1371/journal.pone.0038370 (PMC3366931; doi:10.1371/journal.pone.0038370)
Supplement: Figure S3 — Predicted probabilities for neonatal mortality and infant mortality stratified by maternal anemia. We modeled the distance to a health center as a continuous variable (per increase of 1 km), and used the logarithmic scale (base 2) for the graphs. We averaged the predicted probability for the group less than 1 km from a health center, and showed the probabilities for distance at 1 km increments. (PDF) [file pone.0038370.s007.pdf]

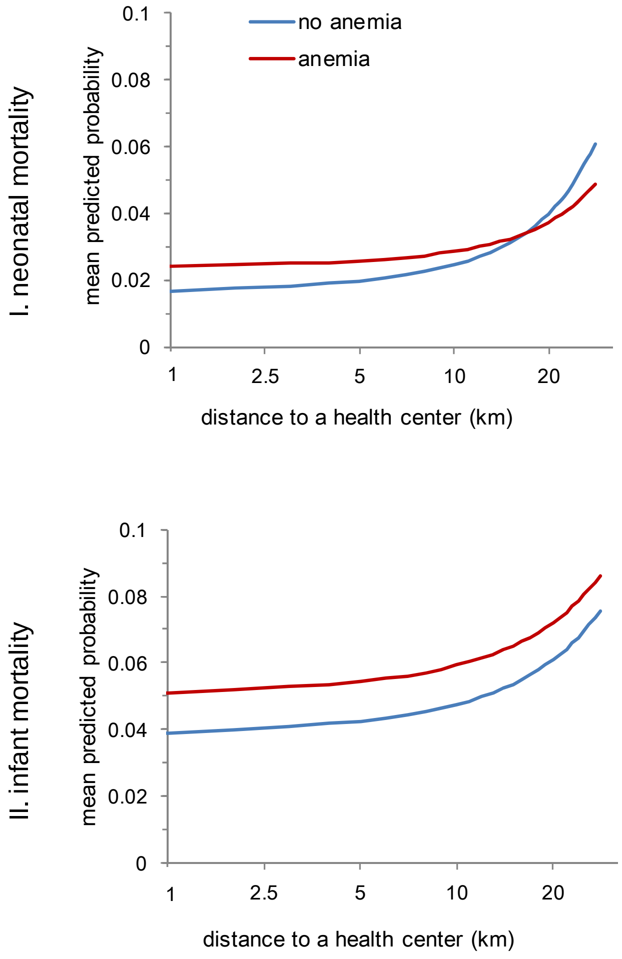

Figure S3. Predicted probabilities for neonatal mortality and infant mortality stratified by maternal anemia.

We modeled the distance to a health center as a continuous variable (per increase of 1 km), and used the logarithmic scale (base 2) for the graphs. We averaged the predicted probability for the group less than 1 km from a health center, and showed the probabilities for distance at 1 km increments.
